# The association of hemodynamic markers of right ventricular dysfunction with SII index and clinical outcomes in reduced ejection fraction heart failure

**DOI:** 10.1097/MD.0000000000034809

**Published:** 2023-08-25

**Authors:** Kevser Balci, İlke Erbay, Burcu Demirkan, Mustafa Mücahit Balci, Ahmet Temizhan

**Affiliations:** a Ankara City Hospital, Ankara, Turkey; b Karabük University, Karabük, Turkey.

**Keywords:** heart failure with reduced ejection fraction, right heart catheterization, systemic immune-inflammation index

## Abstract

Heart failure (HF) is a clinical syndrome with various etiologies and presentations. The role of the inflammatory pathway in HF prognosis is not fully understood. We investigated the association between the systemic immune-inflammation index (SII) and HF complicated by right ventricular dysfunction (RVD) and whether the SII is related to compromised hemodynamic volume status. A total of 235 patients with HF with reduced ejection fraction (HFrEF) were enrolled and divided into 2 groups according to the presence of RVD. The relationship between the SII score, hemodynamic parameters, and clinical endpoints was evaluated. Higher SII scores and neutrophil counts (*P* < .001 and *P* = .017, respectively) were observed in the RVD group (n = 120). In the high SII score group (≥590.4), hospitalization and the need for positive inotrope treatment were significantly higher (*P* = .026 and *P* = .009, respectively), and left ventricular ejection fraction (LVEF) was significantly lower (*P* = .015). In addition, in the high SII score group, right heart catheterization values, including cardiac output and index, were significantly impaired compared with those in the lower SII score group. There was a significant negative correlation between the SII score and the LVEF, cardiac output, and cardiac index in the correlation analyses. A significant relationship was observed between indirect inflammation and RVD in patients with HFrEF. The hemodynamic volume status and functional capacity were impaired in patients with high SII scores. These results indicated that advanced HF with worse outcomes may be related to the inflammatory process.

## 1. Introduction

Heart failure (HF) with reduced ejection fraction (HFrEF) and right ventricular (RV) systolic dysfunction (RVD) are common, associated with clinical and echocardiographic evidence of more advanced HF, and associated with poor prognosis independent of other risk factors.^[1,2]^ Therefore, it is crucial to identify patients who are at high risk of developing RVD.

Currently, several risk-scoring methods have been developed for HF using comorbidities, risk factors, and hematological markers.^[3–6]^ Recently, the systemic immune-inflammation index (SII) was developed based on platelet counts and the neutrophil-to-lymphocyte ratio (N/L) (SII, platelet count × neutrophil/lymphocyte ratio) to simultaneously consider the inflammatory and immune status of patients. Studies have supported an association between high SII and poor outcomes in cancer, coronary artery disease, and HF.^[7–11]^

Although previous evidence has shown that the SII could provide an improved prediction of poor outcomes in patients with HFrEF,^[11]^ to the best of our knowledge, no study has investigated any possible association between the SII and RVD in patients with HFrEF. We aimed to evaluate the association of SII in predicting RVD in patients with HFrEF and whether this association is associated with HF biomarkers, hemodynamic catheterization data, and New York Heart Association (NYHA) class.

## 2. Material and methods

### 2.1. Study population

**Figure 1.**
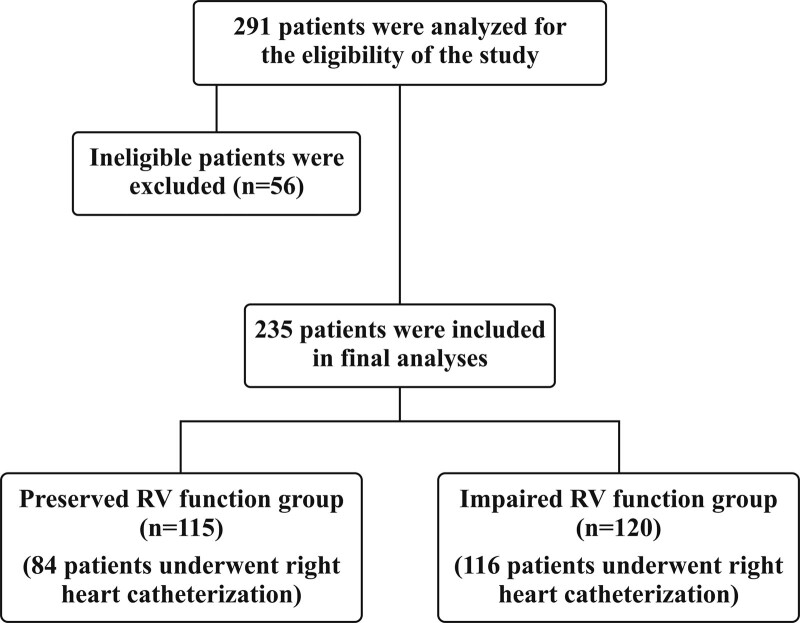
Flowchart of the study.

In the current retrospective observational study, a database of the medical records of 291 consecutive patients in both genders aged between 18 and 80 years with left ventricular ejection fraction (LVEF) < 40 who were admitted to our center between 2010 and 2019 was retrospectively reviewed. Patients with chronic stable HF (no recent diuretic dose increment and/or positive inotrope administration within the last month) and symptoms with a functional NYHA class of I and IV were included in the study (n = 235, female sex n = 36 [15.3%]). Patients with LVEF ≥ 40, recent myocardial infarction or coronary artery bypass graft surgery (≤6 months), decompensated HF, malignancies, chronic inflammatory disease, or hematological disorders were excluded (n = 56) (Fig. 1). Each patient electronic health record was used to obtain information on baseline characteristics, laboratory data, and clinical endpoints. Follow-up of the patients was performed through outpatient visits or phone calls, which were repeated every 6 months. All patients underwent transthoracic echocardiography using a commercially available device (Vivid 7 Ultrasound System; GE Healthcare, Horten, Norway). Patients with echocardiographic signs of RV failure (RV fractional area change < 35% with tricuspid annular systolic velocity <9.5 cm/s or tricuspid annular plane systolic excursion < 17 mm) and findings of RV failure on physical examination were included in the impaired RV function group. Based on the presence of RV dysfunction, the patients were divided into 2 groups: Group 1, preserved RV function group, and Group 2, impaired RV function group. Among them, 116 and 84 patients in the impaired and preserved RV function groups, respectively, underwent right heart catheterization (n = 200). The research protocol was approved by the local Ethics Committee of Ankara City Hospital and followed the principles of the Declaration of Helsinki.

### 2.2. Laboratory parameters and clinical endpoint definition

Peripheral venous blood samples were taken the same day during the patients’ regular checks at outpatient clinics. White blood cell counts with differentials, red blood cell indices, and platelet counts were assessed by an automated blood cell counter (Siemens ADVIA 2120i Hematology System, Siemens Healthcare, Malvern, PA). The SII was calculated as the total peripheral platelet count (P) × N/L (SII = P × N/L ratio). Clinical endpoints were defined as hospitalizations for worsening HF or cardiac death during the follow-up period.

### 2.3. Statistical analyses

Statistical analyses were performed using SPSS software (version 15.0; SPSS Inc., Chicago, IL). Normal distribution was assessed using the Kolmogorov–Smirnov test. Categorical variables are presented as frequencies (n) and percentages (%), and normally distributed variables are presented as mean ± SD. Spearman correlation analysis was also performed. The Kruskal–Wallis test was used for comparisons between more than 2 independent groups. The optimal cutoff points of the SII score to determine RVD were estimated using receiver operating characteristic curve analysis to calculate the area under the curve. Kaplan–Meier survival estimates were calculated. Statistical significance was set at *P* < .05.

## 3. Results

This study included 235 patients with HF, with a median age of 52 years, of whom 36 (15.3%) were women. The median follow-up time was 14 (12–25) months. The baseline characteristics of the study population are presented in Table 1. In group 2 (impaired RV function group), hemoglobin and lymphocyte counts were lower (*P* < .001), and the SII score and neutrophil count were higher (*P* < .001 and *P* = .017, respectively). The need for loop diuretics was significantly higher in group 2 (*P* < .001). Furthermore, the ejection fraction was significantly lower and the left atrial diameter was significantly higher in group 2 (*P* < .001). In addition, deterioration in functional class was more prominent in group 2 (NYHA 3–4 at the 1-year follow-up, 52.2% vs 87.5%, *P* < .001).

**Table 1:** Baseline characteristics of the study population.

Variables	Group 1	Group 2	*P* value
	n: 115	n: 120	
Demographic and clinical characteristics			
Age, yr	52 (21–77)	52 (19–70)	.870
Female gender, n (%)	18 (15.7%)	18 (15.0%)	.890
Hypertension, n (%)	59 (51.3%)	51 (42.5%)	.176
Diabetes mellitus, n (%)	35 (30.4%)	41 (34.2%)	.541
Ischemic HF, n (%)	57 (49.6%)	50 (41.7%)	.224
Atrial Fibrillation, n (%)	21 (18.3%)	32 (26.7%)	.123
ICD/CRT, n (%)	90 (78.3%)	109 (90.8%)	.007
Baseline NYHA 3–4, n (%)	12 (10.4%)	65 (54.2%)	<.001
Laboratory parameters			
eGFR, mL/min	86 (34–194)	80 (12–161)	.016
Uric acid, mg/dL	6.3 (1.4–15.1)	7.4 (3.4–15.6)	<.001
Sodium, mEq/L	140 (110–147)	138 (123–147)	<.001
Potassium, mEq/L	4.6 (3.5–5.8)	4.4 (2.9–5.9)	.026
AST, U/L	21 (11–479)	24 (9–2167)	<.001
ALT, U/L	24 (5–551)	23 (9–2743)	.479
GGT, mg/dL	31 (10–632)	77 (15–589)	<.001
LDH, mg/dL	211 (127–680)	279 (121–4807)	<.001
Direct bilirubin, mg/dL	0.22 (0.07–2.27)	0.53 (0.1–15.7)	<.001
Indirect bilirubin, mg/dL	0.44 (0.01–1.68)	0.65 (0.09–23.0)	<.001
Albumin, mg/dL	45 (30–52)	41 (25–52)	<.001
NT-proBNP, pg/mL	881 (33–25000)	5728 (213–35000)	<.001
Medications, n (%)			
Beta-blocker	114 (99.1%)	116 (96.7%)	.370
ACEi/ARB/ARNI	115 (100%)	113 (94.2%)	.014
Digoxin	15 (13.0%)	21 (17.5%)	.343
MRAs	99 (86.1%)	100 (83.3%)	.558
Loop diuretics	68 (59.1%)	99 (82.5%)	<.001
Ivabradin	11 (9.6%)	7 (5.8%)	.282
Hematological parameters			
Hemoglobin, g/dL	14.5 (10.0–15.1)	13.4 (8.3–17.7)	<.001
Platelets, ×10^3/µL	228 (114–471)	234 (94–600)	.569
WBC, ×10^3/µL	8.0 ± 1.9	8.9 ± 3.3	.220
Neutrophil, ×10^3/µL	4.7 (2.3–11.3)	5.4 (2.4–20.7)	.017
Lymphocyte, ×10^3/µL	2.0 (0.8–3.9)	1.6 (0.2–12.8)	<.001
SII score	536.8 (146.4–1986.3)	720.3 (128.7–23017.5)	<.001
Echocardiographic characteristics			
EF, %	28 (15–39)	20 (10–35)	<.001
LVEDD, mm	62 (47–94)	65 (41–105)	.014
LA, mm	44 (40–72)	50 (35–101)	<.001
E/E’	12 (5.5–18)	11.5 (6–18)	.871
SPAP, mm Hg	35 (20–65)	45 (22–85)	<.001
TAPSE, mm	20 (9–26)	13 (7–23)	<.001
RV fractional area change	45 (37–55)	27 (15–34)	<.001
Tricuspid annular systolic velocity	11 (10–14)	7 (4.5–9)	<.001
Clinical end points at 1-yr follow-up			
Hospitalization number	1 (0–4)	2 (0–5)	<.001
Need for positive inotropes at hospitalization, n (%)	27 (27.8%)	70 (72.2%)	<.001
Death, n (%)	0 (0%)	23 (19.2%)	<.001
NYHA 3–4 at 1-yr follow-up, n (%)	60 (52.2%)	105 (87.5%)	<.001

ACEi = angiotensin-converting-enzyme inhibitors, ALT = alanine aminotransferase, ARB = angiotensin II receptor blocker, ARNI = angiotensin receptor-neprilysin inhibitor, AST = aspartate aminotransferase, CRT = cardiac resynchronization therapy, EF = ejection fraction, eGFR = estimated glomerular filtration rate, GGT = gamma-glutamyl transferase, HF = heart failure, ICD = implantable cardioverter-defibrillator, LA = left atrium, LDH = lactate dehydrogenase, LVEDD = left ventricular end diastolic diameter, MRA = mineralocorticoid receptor antagonist, NT-proBNP = N-terminal pro-B-type natriuretic peptide, NYHA = New York heart association class, SII = systemic immune-inflammation index, SPAP = systolic pulmonary artery pressure, TAPSE = tricuspid annular plane systolic excursion, WBC = White blood cell count.

Correlation analysis was performed to determine the relationship between the SII score and N-terminal probrain natriuretic peptide levels, and a significant correlation was found between the SII score and N-terminal probrain natriuretic peptide levels in all patients (rho = 0.324, *P* < .001). The SII score was significantly negatively correlated with LVEF, RV fractional area change, tricuspid annular plane systolic excursion, cardiac output, and cardiac index (Table 2). In addition, the SII score was positively correlated with right atrial pressure and systolic pulmonary artery pressure.

**Table 2:** Correlation analyses between SII score and left and right ventricle ejection fraction and hemodynamic parameters.

	LVEF	RVFAC	TAPSE	RAP	SPAP	MPAP	PCWP	CO	CI	PVR
SII score	r = −0.148	r = −0.281	r = −0.274	*R* = 0.281	*R* = 0.263	*R* = 0.270	*R* = 0.161	r = −0.230	r = −0.213	*R* = 0.370
	*P* = .024	*P* < .001	*P* < .001	*P* < .001	*P* < .001	*P* < .001	*P* = .022	*P* = .001	*P* = .002	*P* < .001

CI = cardiac index, CO = cardiac output, LVEF = left ventricular ejection fraction, MPAP = mean pulmonary artery pressure, PCWP = pulmonary capillary wedge pressure, PVR = pulmonary vascular resistance, RAP = right atrial pressure; RVFAC; right ventricular fractional area change, SII = systemic immune-inflammation index, SPAP = systolic pulmonary artery pressure, TAPSE = tricuspid annular plane systolic excursion.

Between the baseline NYHA functional classes, there was a significant difference in the SII score; the SII increased as the NYHA functional class worsened (Table 3). Furthermore, the high SII score group had significantly impaired right heart catheterization values, including cardiac output and cardiac index, compared to the lower SII score group (Table 4).

**Table 3:** Evaluation of the possible prognostic factors in HF according to the baseline functional classes.

Variables	NYHA 1	NYHA 2	NYHA 3	NYHA 4	*P* value
	n = 55	n = 103	n = 66	n = 11	
EF, %	31 (15–39) ^a,b,c^	20 (10–39) ^a,d^	20 (10–23) ^b,d^	20 (10–20) ^c^	<.001φ
Impaired RV functions, n (%)	10 (18.2%) ^a,b,c^	45 (43.7%) ^a,d,e^	54 (81.8%) ^b,d^	11 (100%) ^c,e^	<.001¶
NT-proBNP	455 (33–7976) ^a,b,c^	1970 (35–14823) ^a,d,e^	9198 (547–35000) ^b,d,f^	15860 (1420–35000) ^c,e,f^	<.001φ
SII	497.1 (249.7–10468.6) ^b,c^	589.2 (128.7–5655.9) ^d^	804.5 (164.7–6994.3) ^b,d^	927.0 (447–823017.5) ^c^	<.001φ
Hospitalization at follow-up, n (%)	20 (36.4%) ^a,b,c^	82 (79.6%) ^a,d^	64 (97.0%) ^b,d^	11 (100%) ^c^	<.001¶
Death at follow-up, n (%)	0 (0%) ^b,c^	1 (1%) ^d,e^	14 (21.2%) ^b,d,f^	8 (72.7%) ^c,e,f^	<.001¶

¶ Likelihood Ratio test,

φ Kruskal-Wallis test: According to Bonferroni adjustment, a value of *P* < .012 was accepted as significant.

a: The difference between NYHA 1 and NYHA 2 groups is significant,

b: Difference between NYHA 1 and NYHA 3 groups is significant,

c: Difference between NYHA 1 and NYHA 4 groups is significant,

d: Difference between NYHA 2 and NYHA 3 groups is significant,

e: Difference between NYHA 2 and NYHA 4 groups is significant,

f: Difference between NYHA 3 and NYHA 4 groups is significant,

EF = ejection fraction; NT-proBNP = N-terminal pro-B-type natriuretic peptide; NYHA = New York Heart Association class; RV = right ventricle; SII = Systemic immune-inflammation index.

**Table 4:** Evaluation of the right heart catheterization according to the SII score.

Variables	SII < 590.4	SII ≥ 590.4	*P* value
Right atrial pressure, mm Hg	8 (3–28)	10 (3–27)	.001
Systolic pulmonary artery pressure, mm Hg	44.4 ± 16.5	51.9 ± 15.6	.001
Diastolic pulmonary artery pressure, mm Hg	19.5 (5–48)	24 (2–40)	<.001
Mean pulmonary artery pressure, mm Hg	28.5 (10–71)	35 (8–55)	<.001
Pulmonary capillary wedge pressure, mm Hg	20 (6–40)	22 (7–43)	.014
Cardiac output, L/min	3.9 (1.8–9.3)	3.1 (1.6–8.4)	.002
Cardiac index, L/min	2.0 (1.0–4.9)	1.7 (0.9–4.5)	.002
Pulmonary vascular resistance, mm Hg/L/min	2.0 (1.0–14)	3 (1.0–13)	<.001

SII = systemic immune-inflammation index.

**Figure 2.**
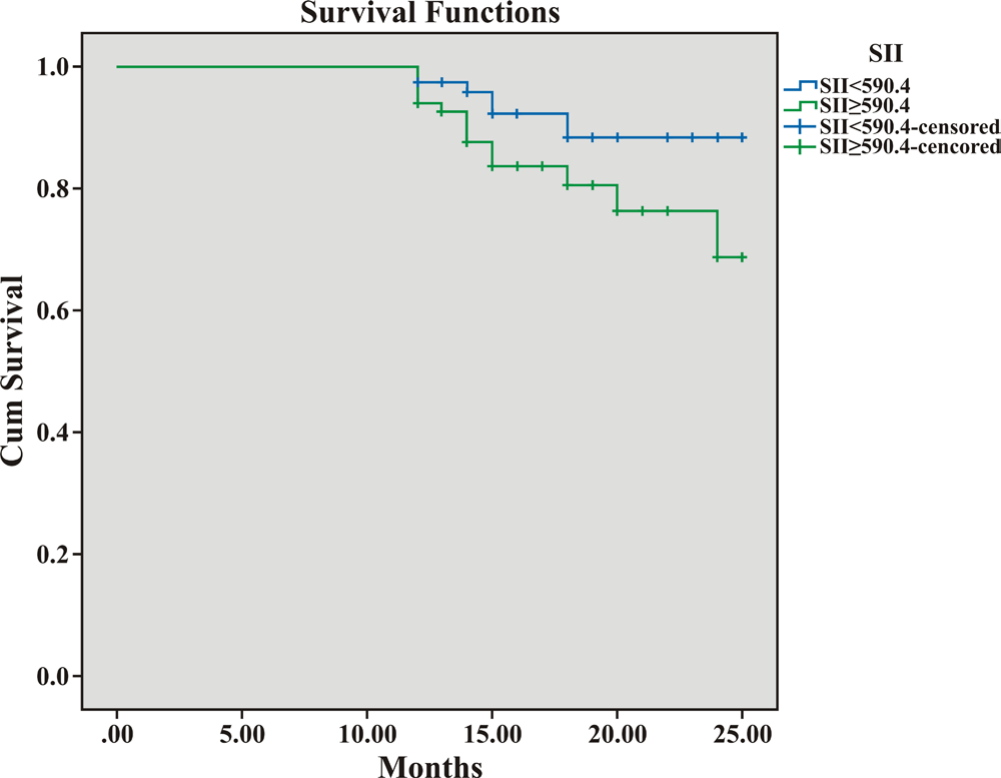
Survival curve of the patients according to the high SII score (*P* = .052). SII = systemic immune-inflammation index.

Receiver operating characteristic curve analysis showed that an SII of 590.4 was a fair discriminator for RVD with a sensitivity of 61.7% and a specificity of 60.9% (area under the curve: 0.648, 95% confidence interval: 0.578–0.719, *P* < .001). When clinical endpoints and LVEF were evaluated for a high SII score (≥590.4), hospitalization rates at the 1-year follow-up and the need for positive inotropes at hospitalization were significantly higher, and LVEF was significantly lower in the high SII score group (*P* = .026 and *P* = .009, respectively) (Table 5). When medications were analyzed according to high SII scores, only loop diuretic use was significantly higher in the high SII score group (64.7% vs 77.3%, *P* = .032). However, the Kaplan–Meier survival analysis showed that death during follow-up did not change according to a high SII score (*P* = .052) (Fig. 2).

**Table 5:** Clinical end points and LVEF according to the SII score.

Variables	SII < 590.4	SII ≥ 590.4	*P* value
Hospitalization for HF, n (%)	80 (69%)	97 (81.5%)	.026
Need for positive inotropes, n (%)	38 (32.8%)	59 (49.6%)	.009
Death at 1-yr follow-up, n (%)	7 (6%)	16 (13.4%)	.056
LVEF, %	25 (10–39)	20 (10–39)	.015

HF = heart failure, LVEF = left ventricular ejection fraction, SII = systemic immune-inflammation index.

## 4. Discussion

The major findings of our single-center retrospective study showed that a higher SII was associated with RVD and a higher NYHA class and that the SII was positively correlated with NTpro-BNP levels as a marker of HF. A higher SII score was associated with lower LVEF. Regarding clinical endpoints, a higher SII score was associated with the risk of rehospitalization and the need for inotropic support for decompensated HF. In addition, RVD was more pronounced in the high SII group as confirmed by right heart catheterization. To the best of our knowledge, this is the first report on the potential role of the SII as a discriminator of RVD in patients with HF.

RVD is independently correlated with mortality and hospitalization due to the risk of HF in left ventricular failure.^[12]^ Despite the exact pathophysiology, the cause of the underlying RVD remains unclear. However, accumulating evidence suggests that triggers such as pressure or volume overload or myocardial diseases such as RV infarction or cardiomyopathy contribute to the activation of inflammatory pathways.^[13]^ Moreover, Harhay et al demonstrated in an interesting magnetic resonance imaging investigation that plasma levels of pro-inflammatory C-reactive protein and interleukin-6 were independently associated with RV morphology, even in individuals without cardiovascular disease.^[14]^

SII, a novel noninvasive biomarker based on 3 peripheral blood parameters (platelet, neutrophil, and lymphocyte counts), can effectively reflect the balance between the host immune and inflammatory status.^[12]^ Inflammation plays a vital role in cardiovascular disease. The interplay between leukocyte differentials and HF is complex; systemic release of cytokines, activation of the hypothalamic–pituitary–adrenal axis, and neutrophils are responsible for disease progression and mortality.^[15–17]^ In agreement with this, Yang et al showed that a higher SII is independently associated with a higher risk of future cardiac death, nonfatal MI, nonfatal stroke, or hospitalization for HF among patients with coronary artery disease.^[10]^ Seo et al also reported that increased SII scores were independently associated with mortality in patients with HF.^[11]^ Unlike previous reports, we did not observe higher mortality in the Kaplan–Meier survival analysis. However, the *P* value for the mortality difference according to the high SII score was 0.052, which might indicate a trend toward significance for mortality. In our study, we observed that higher SII scores were related to advanced disease; the SII score in the RVD group was significantly higher than that in the preserved RV function group. In addition, the LVEF was significantly lower in the high SII score group, indicating a relationship between inflammation and disease progression. Inflammation, a possible stress response, may be more pronounced in advanced HF and could explain the lower LVEF in the high SII score group. Previously, Wojciechowska et al demonstrated more active inflammation in patients with dilated cardiomyopathy who were in NYHA classes 3 to 4 than in patients who were in NYHA classes 1 to 2.^[18]^ We also found that NYHA functional capacity deteriorated in correlation with an increase in the SII scores. A previous study reported a relationship between worse functional capacity and inflammation which was linked to non-response to cardiac resynchronization therapy.^[19]^ Therefore, the authors concluded that the inflammation status was related to disease severity. Regarding the clinical endpoints, we showed that in the high SII score group, the incidence of hospitalization during a 1-year follow-up and the need for positive inotropes at hospitalization were significantly higher. Therefore, our findings support the harmful effects of inflammation on HF progression and prognosis.^[15–17]^

In patients with HF, the hemodynamic volume status is modifiable with medications, and a deteriorated volume status is related to pulmonary and systemic congestion, which results in worse functional capacity. There is a relationship between systemic inflammation and RV failure.^[20]^ Wojciechowska et al showed a relationship between inflammation and systemic and pulmonary hemodynamic parameters; serum activities of superoxide dismutase isoenzymes were significantly correlated with pulmonary capillary wedge pressure, mean pulmonary artery pressure, and LVEF.^[18]^ Similarly, we showed that the SII score is associated with both LVEF and invasively derived hemodynamic parameters. This indicates that indirect inflammation parameters not only show advanced disease, but are also informative regarding hemodynamic volume status.

The current study has some limitations. This was a single-center, retrospective study with a small sample size. Inherent to the retrospective design, the patients were not randomized regarding their demographic properties. Additionally, further inflammation markers, including mean platelet volume possibly related to HF progression, were not evaluated, and follow-up time was relatively short.

## 5. Conclusions

In patients with HFrEF, a higher SII score was associated with lower LVEF, RVD, and hemodynamically confirmed advanced disease. Among patients with a higher SII score, the incidence of hospitalization and need for positive inotrope support during decompensation were higher. In addition, a higher SII score was associated with deterioration in hemodynamic volume status and functional capacity. These findings may indicate neurohumoral activation of the subject and a stress response to HF owing to the inflammatory process.

## Acknowledgments

The data of the patients enrolled in the present study were also included in the Master thesis of Doctor İlke Erbay.

## Author contributions

**Data curation:** İlke Erbay, Mustafa Balci.

**Investigation:** Kevser Balci, İlke Erbay, Burcu Demirkan.

**Methodology:** Kevser Balci, Mustafa Balci.

**Supervision:** Burcu Demirkan, Ahmet Temizhan.

**Writing – review & editing:** Kevser Balci.
